# Home Respiratory Polygraphy to Detect Obstructive Sleep Apnea Syndrome after Supracricoid Partial Laryngectomy

**DOI:** 10.1055/s-0044-1801791

**Published:** 2025-01-23

**Authors:** Rosa Hernández-Sandemetrio, Natsuki Oishi, Tomás Chavero, Rafael Navarro, Isabel López, Enrique Zapater

**Affiliations:** 1 ENT Department, University General Hospital of Valencia, Valencia School of Medicine, Valencia, Spain; 2 Intensive Care Unit Department, University Doctor Peset Hospital, Valencia, Spain; 3 Pneumology Department, University General Hospital of Valencia, Valencia, Spain

**Keywords:** obstructive sleep apnea, partial laryngectomy, polygraphy

## Abstract

**Introduction** Supracricoid partial laryngectomy is a surgical treatment for advanced laryngeal cancer which is implemented to preserve organ function, but it may cause obstructive sleep apnea syndrome (OSAS) due to anatomical changes after surgery that may be neglected by clinicians.

Although the gold standard for the diagnosis of OSAS is polysomnography, respiratory polygraphy is an alternative valid method with a high level of diagnostic sensitivity and specificity; since the equipment is portable, it can be used at home, with no need for hospitalization.

**Objective** To describe the polygraphy result of patients submitted to supracricoid partial laryngectomy.

**Methods** The present study included 13 patients, and we collected data on age, date of the surgery, body mass index, cardiovascular risk factors, Epworth score, and apnea-hypopnea index (AHI).

**Results** The 13 patients were all male, with a mean age of 62 years. As for the AHI, one patient was classified as severe, six, as moderate, and three patients, as mild; moreover, 3 patients were simple snorers. While 77% of the sample presented OSAS, only 23% presented symptoms of drowsiness.

**Conclusion** The study group, who underwent supracricoid partial laryngectomy, did not present self-reported symptoms of OSAS. Nevertheless, polygraphy was a useful tool in this group, and we recommend its systematic use after decannulation to avoid leaving OSAS undiagnosed.

**Level of evidence** 4; case series study

## Introduction

Supracricoid partial laryngectomy (SPL) is a surgical treatment for advanced laryngeal cancer which is implemented to preserve organ function. 1 2 3 The incidence of obstructive sleep apnea syndrome (OSAS) in patients undergoing SPL is high due to anatomical changes that occur after surgery. 2 3 4 5 Postoperatively, the larynx can suffer collapses, causing microarousal and sleep fragmentation, even though these patients do not usually report diurnal drowsiness. Indeed, complaints related to sleepiness and snoring may be neglected by clinicians in the context of cancer surgery; nonetheless, the possibility of developing OSAS after the completion of a partial laryngectomy must be kept in mind. 5

The gold standard for the diagnosis of OSAS is polysomnography performed while asleep at a hospital sleep laboratory, which thereby requires hospitalization. 6 The apnea–hypopnea index (AHI) determines the severity of this syndrome and its corresponding increased cardiovascular risk. Respiratory polygraphy is an alternative valid method with a high level of diagnostic sensitivity and specificity, 7 which can be used at home through a portable equipment.

The aim of the present study was to describe the polygraphy results of patients submitted to SPL.

## Methods

The inclusion criteria were patients who underwent SPL at our institution from 2009 to 2019, and the exclusion criteria were patients who were non-decannulated, not able to understand the instructions, who lacked family support, required continuous positive airway pressure, with active oncological disease or severe cardiovascular illness, or who had deceased.

We included 13 patients, who signed the informed consent form to participate. The institutional Ethics Committee approved the study (registration: 124/2021). All patients received practical information on polygraph management. A portable polygraph device (Alice PDx, Philips Respironics, Murrysville, PA, United States) was used so patients could perform the examination at home, measuring nasobucal airflow, heart rate, arterial oxygen saturation, respiratory effort, body position, and snoring.

A database was created in which we collected data on age, surgery date, body mass index (BMI), cardiovascular risk factors (such as smoking, high blood pressure, and dyslipidemia), the Epworth score, the date of the polygraphy, and the AHI. The diagnosis of OSAS was established according to the AHI as mild (score between 10 and 15), moderate (score ranging from 16 to 29), or severe (score ≥ 30). We also used the Epworth scale, a self-administered questionnaire, to evaluate daytime hypersomnia, with scores exceeding 10 being pathological. 8

The statistical analyses were all conducted using the IBM SPSS Statistics for Windows software, version 24.0, (IBM Corp., Armonk, NY, United States). Basic descriptive data were obtained for each variable and were correlated with the AHI score.

## Results

All of the patients in the present series were male, with a mean age of 62 years, and their cardiovascular risk factors were as follows: obesity (the mean BMI was of 27 Kg/m ^2^ and 61% of the patients presented a BMI > 25 Kg/m ^2^ ), smoking (15%), diabetes (15%), high blood pressure (38%), and dyslipidemia (31%).

In terms of the polygraph data, the mean recording time was of 402.88 (minimum: 179) minutes, with a standard deviation (SD) of ± 138 minutes. The average number of respiratory events registered during the recording was of 114 (SD: ± 69; minimum: 39; maximum: 247).

**Fig. 1:**
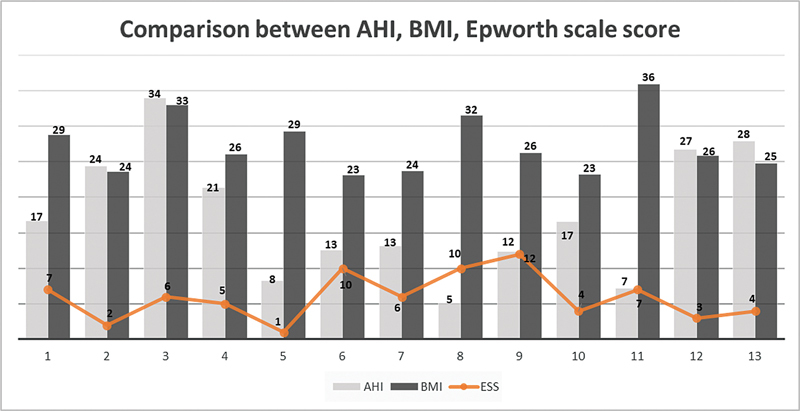
Comparison regarding the AHI, the BMI, and the score on the Epworth scale.

The AHI was at a pathological level in most patients: one patient was classified as severe, six, as moderate, and three, as mild, while three patients were classified as simple snorers ( Fig. 1 ). While 77% presented OSAS, only 23% presented symptoms of drowsiness according to the Epworth scale, even patients diagnosed with severe or moderate OSAS ( Fig. 1 ).

We did not observe a direct correlation between BMI and apnea–hypopnea episodes. In total, 3 patients were obese (BMI > 30 Kg/m ^2^ ), and only 1 of them presented OSAS. Among the patients with severe OSAS, only 1 was obese;, the other 2 were overweight, with BMIs of 24 Kg/m ^2^ and 25 Kg/m ^2^ respectively ( Fig. 1 ).

## Discussion

In the present study, 77% of the patients were diagnosed with OSAS according to the respiratory polygraphy results, and most were mild-to-moderate cases. This finding is similar to those of other studies using polysomnography for the diagnosis, 2 3 4 and it suggests that the prevalence of OSAS is increased in patients submitted to surgery for larynx cancer in comparison to the general population.

Self-reported sleep symptoms alone may be unreliable to determine the risk of OSAS in the population of patients with larynx cancer treated either by surgery or radiotherapy. 9 10 These patients may suffer fatigue, but this symptom is often misdiagnosed because of their base illness. In the present study, the scores on the Epworth scale were not consistent with the diagnosis of OSAS. We also found that the BMIs of the patients were not directly correlated with the AHI, which could be explained by postoperative anatomical changes, suggesting that this population may be distinct.

The results of the present study imply that polygraphy screening among larynx cancer patients treated by partial laryngectomy would be beneficial. The advantage of this approach compared to polysomnography is that these tests can be performed at home, and they are accessible and cost-effective. For illustration purposes, in our care system, a polysomnogram costs €843.74, while a respiratory polygraph costs €141.67. In addition, avoiding hospitalization is extremely useful, especially in times such as the coronavirus disease 2019 (Covid-19) pandemic. As a last note, the polygraph must be interpreted in a clinical context, and in case of discordance or doubt, a polysomnogram should be performed.

## Conclusion

The patient population that underwent SPL did not present self-reported symptoms of OSAS. Nonetheless, OSAS screening using polygraphy was a useful tool in this group, and we recommended its systematic use after decannulation to avoid leaving OSAS undiagnosed.
